# Cytoprotective Effects of Human Platelet Lysate during the Xeno-Free Culture of Human Donor Corneas

**DOI:** 10.3390/ijms24032882

**Published:** 2023-02-02

**Authors:** Delia Talpan, Sabine Salla, Linus Meusel, Peter Walter, Chao-Chung Kuo, Julia Franzen, Matthias Fuest

**Affiliations:** 1 Department of Ophthalmology, RWTH Aachen University, 52074 Aachen, Germany; 2 Cornea Bank Aachen, RWTH Aachen University, 52074 Aachen, Germany; 3 Genomics Facility, Interdisciplinary Center for Clinical Research (IZKF), RWTH Aachen University, 52074 Aachen, Germany

**Keywords:** cornea, culture, fetal calf serum, fetal bovine serum, platelet lysate

## Abstract

We evaluated the suitability of 2% human platelet lysate medium (2%HPL) as a replacement for 2% fetal bovine serum medium (2%FBS) for the xeno-free organ culture of human donor corneas. A total of 32 corneas from 16 human donors were cultured in 2%FBS for 3 days (TP1), then evaluated using phase contrast microscopy (endothelial cell density (ECD) and cell morphology). Following an additional 25-day culture period (TP2) in either 2%FBS or 2%HPL, the pairs were again compared using microscopy; then stroma and Descemet membrane/endothelium (DmE) were processed for next generation sequencing (NGS). At TP2 the ECD was higher in the 2%HPL group (2179 ± 288 cells/mm^2^) compared to 2%FBS (2113 ± 331 cells/mm^2^; *p* = 0.03), and endothelial cell loss was lower (ECL HPL = −0.7% vs. FBS = −3.8%; *p* = 0.01). There were no significant differences in cell morphology between TP1 and 2, or between 2%HPL and 2%FBS. NGS showed the differential expression of 1644 genes in endothelial cells and 217 genes in stromal cells. It was found that 2%HPL led to the upregulation of cytoprotective, anti-inflammatory and anti-fibrotic genes (HMOX1, SERPINE1, ANGPTL4, LEFTY2, GADD45B, PLIN2, PTX3, GFRA1/2), and the downregulation of pro-inflammatory/apoptotic genes (e.g., CXCL14, SIK1B, PLK5, PPP2R3B, FABP5, MAL, GATA3). 2%HPL is a suitable xeno-free substitution for 2%FBS in human cornea organ culture, inducing less ECL and producing potentially beneficial alterations in gene expression.

## 1. Introduction

Transplantation remains an integral treatment for advanced corneal disease [1,2], despite the groundbreaking developments in corneal endothelial cell culture and therapy seen in recent years [3]. New lamellar keratoplasty techniques have improved graft survival and patient outcome [4,5,6]. For instance, in Germany, the number of corneal transplantations has increased by 1.5-fold, from 4730 penetrating keratoplasties (PKP) in 2001 to 7325 penetrating and lamellar keratoplasties in 2016 [7]. The tissue is usually provided through specialized eye banks, which store the corneoscleral button in either hypothermic (2–6 °C, e.g., USA) or organ culture (31–37 °C) conditions [8]. Hypothermic storage is less complicated and cheaper. In general, a pre-storage evaluation of the endothelium is performed by specular microscopy and storage time is usually around 7–10 days [8].

Organ culture is a relatively sophisticated technique requiring more expertise and well-equipped facilities. Evaluation of the cornea is not only performed at the start and at the end, but is also possible throughout storage via light microscopy, which allows for the monitoring of the transplants and endothelial cells for pathologic alterations, contamination and endothelial cell loss (ECL), enhancing quality control and safety [9,10,11]. The organ culture storage period is longer; up to four weeks. The susceptibility of organ culture to microbial contamination can be turned into an advantage because it allows the detection of remaining micro-organisms on the cornea before surgery [9]. Both preservation techniques seem to result in similar graft survival [8].

Current organ culture protocols contain fetal bovine serum (FBS) in concentrations ranging from 2–8% [12]. Nevertheless, comprehensible concerns have been raised regarding the safety of FBS-based culture media. Bovine antigens, for instance, accumulate intracellularly, hence, cells expanded in FBS-containing medium can cause anaphylactic reactions if administered repeatedly [13,14,15,16]. The ingredients of FBS are not precisely defined and there is a high lot-to-lot variation [17]. FBS can contain high endotoxin levels, potentially inducing the production of proinflammatory and profibrogenic cytokines in cultured cells [17,18,19]. FBS concentrations higher than 0.5% can lead to the conversion of corneal stromal keratocytes (CSK) into stromal fibroblasts, and further into scar-inducing myofibroblasts (Myo-SF) [19,20,21]. Additionally, the bleeding procedure of bovine fetuses necessary for FBS production is of animal welfare concern [17]. Cell treatments and organ preservation for clinical application should ideally be carried out according to current good manufacturing practices (cGMP) [22]. GMP covers all aspects of the manufacturing process, including raw material selection and qualification. It emphasizes that all materials that come in direct contact with cell therapy products should be of appropriate quality. Additionally, there are more specific considerations for animal-sourced products [23,24]. Hence, to increase the safety of cell therapy products and transplants, most regulatory agencies recommend omitting animal-derived materials in the culture process if possible [22,23,24,25,26].

Human platelet lysate (HPL) could be a viable alternative to FBS. HPL is easily generated by a freeze–thaw procedure of human platelet concentrates after expiration [17,27,28]. Being allogenic, there is no risk of xenogenic immune reactions or the transmission of bovine pathogens [17]. Furthermore, HPL can be used in autologous settings to further reduce risks of contamination or immune reactions [17].

**Figure 1:**
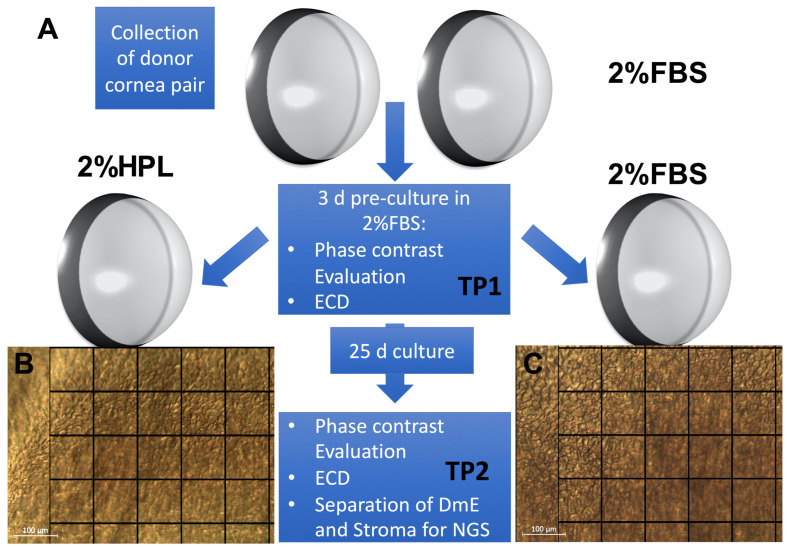
(**A**) Experimental workflow. The 32 corneas (16 pairs) from 16 donors were collected using corneoscleral excision and pre-cultured in 2% FBS medium (2%FBS). After 3 days (TP1) and evaluation of cell density and morphology, the pairs were split up into either 2%FBS or 2% HPL medium (2%HPL). After 25 days of differential culture conditions (time point TP2), phase contrast evaluation (donor 3 showing a healthy corneal endothelium layer in 2%HPL (**B**) and 2%FBS (**C**), a square equals 100 × 100 µm) was repeated, stroma and Descemet membrane/endothelium (DmE) were separated and processed for next generation sequencing (NGS, 15 pairs).

In this study we evaluated the suitability of 2% HPL containing medium (2%HPL) vs. 2% FBS medium (2%FBS) for the organ culture of 16 cornea donor pairs (Table 1 and Figure 1).

## 2. Results

### 2.1. 2%HPL Led to a Lower Endothelial Cell Loss Than 2%FBS

ECD did not differ between the 2%HPL and 2%FBS group at TP1 (*p* = 0.87, Table 2). At TP2 the ECD was significantly higher in the 2%HPL group (2179 ± 288 cells /mm^2^) compared to 2%FBS (2113 ± 331 cells /mm^2^; *p* = 0.03), and endothelial cell loss (ECL) was significantly lower (HPL = −0.7% vs. FBS= −3.8%; *p* = 0.01).

### 2.2. 2%HPL and 2%FBS Did Not Influence Endothelial Cell Morphology

Overall, the corneal endothelial cells (CECs) showed only mild alterations at TP1 and TP2, with no trypan blue positive cells at any time. There were no significant differences in polymegethism, pleomorphism, granulation, vacuolization, segmentation of cell membranes, Descemet folds, or endothelial cell-free areas, neither between TP1 and 2 nor between groups (Table 3).

### 2.3. NGS Indicated a More Extensive and Robust Differential Gene Regulation in Endothelial Compared to Stromal Corneal Cells

**Figure 2:**
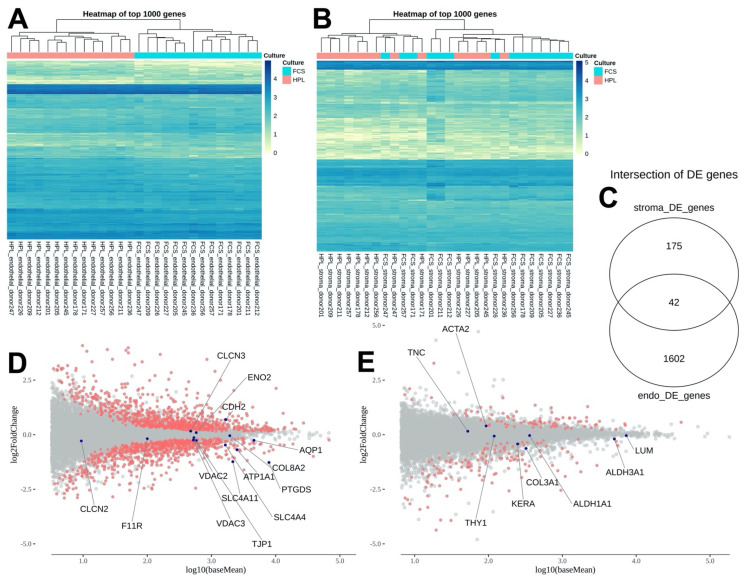
Next generation sequencing data of the stroma and Descemet membrane/endothelium (DmE) complex of the donor corneas after 25 days of differential culture conditions in either 2% HPL or 2% FBS-containing medium. Heat-maps of the top 1000 genes ranked by adjusted *p*-value comparing the differential gene expression patterns of the DmE (**A**) and stroma (**B**). In total, 1602 genes were differentially regulated in the DmE and 175 in the stroma, while 42 were differentially regulated in the stroma as well as the DmE (**C**). MAplot of the differences in the expression of known marker genes for corneal endothelial (**D**) and stromal cells (**E**).

Looking at the endothelial and stromal cells individually, we found that 25 days of culture in either 2%FBS or 2%HPL led to the differential expression of 1644 genes in the CECs (Figure 2A,C) and 217 genes in the corneal stromal cells (CSKs, Figure 2B,C). The dendrograms of hierarchical clustering (Figure 2A,B) show that the expression changes are consistent among the DmE samples, while there are some outliers among the stromal samples. The top 10 up- and downregulated endothelial and stromal genes can be found in Table 4. They are further characterized, focusing on their relevance to the cornea and organ culture, in Supplementary Data S3.

### 2.4. Differential 2%HPL and 2%FBS Culture Did Not Influence the Expression Levels of Known Corneal Stromal Cell Markers, but Caused Mild Alterations among Corneal Endothelial Cell Markers

There were no significant differences in gene expression levels for corneal stromal cell marker genes (Table 5). Five corneal endothelial cell marker genes were differentially regulated when cultured in either 2%HPL or 2%FBS for 25 days. PTGDS, ATPA1, SLC4A11, SLC4A4 were downregulated in 2%HPL compared to 2%FBS, while CDH2 was upregulated in 2%HPL. However, the log2FCs only ranged from −1.24 to 0.69, which indicates only a slight difference (Table 5; Figure 2D,E).

### 2.5. Category Netplots Visualize That Gene Expression Alterations following 2%HPL vs. 2%FBS Cornea Culture Influenced Different Areas of Cell Function in Stromal Compared to Corneal Endothelial Cells

**Figure 3:**
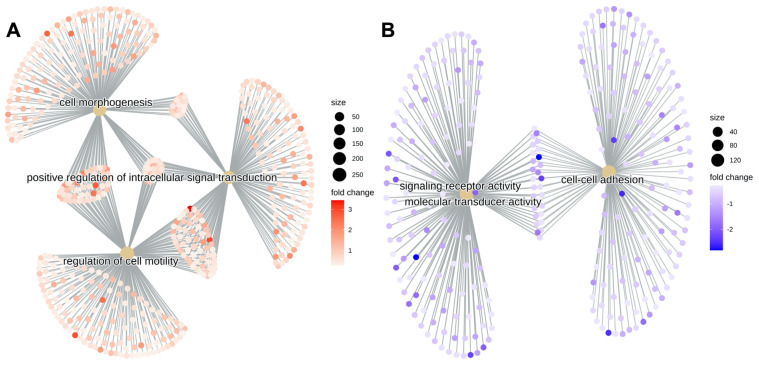
Category netplots depicting the linkages of differentially regulated genes and biological concepts (GO terms and KEGG pathways) as a network extracted from the next generation sequencing data of the Descemet membrane/endothelium complex (DmE, (**A**) and the stroma (**B**) of the donor corneas after 25 days of differential culture conditions in either 2%HPL or 2%FBS.

By grouping the GO terms into category netplots, and depicting the linkages between genes and biological concepts as a network, we found that in the DmE complex, genes that were differentially regulated between the 2%HPL and 2%FBS corneas accumulated in the areas of cell morphogenesis, positive regulation of intracellular signal transduction and regulation of cell motility (Figure 3A), while in the stroma the alterations occurred mainly in the areas of signal receptor activity, molecular transducer activity and cell–cell adhesion (Figure 3B).

### 2.6. GSEA (Gene Set Enrichment Analysis) of Hallmark Gene Sets and Associated DGEA (Differential Gene Expression Analysis) Showed Differential Expression Patterns Predominantly in the Corneal Endothelial Cells

**Figure 4:**
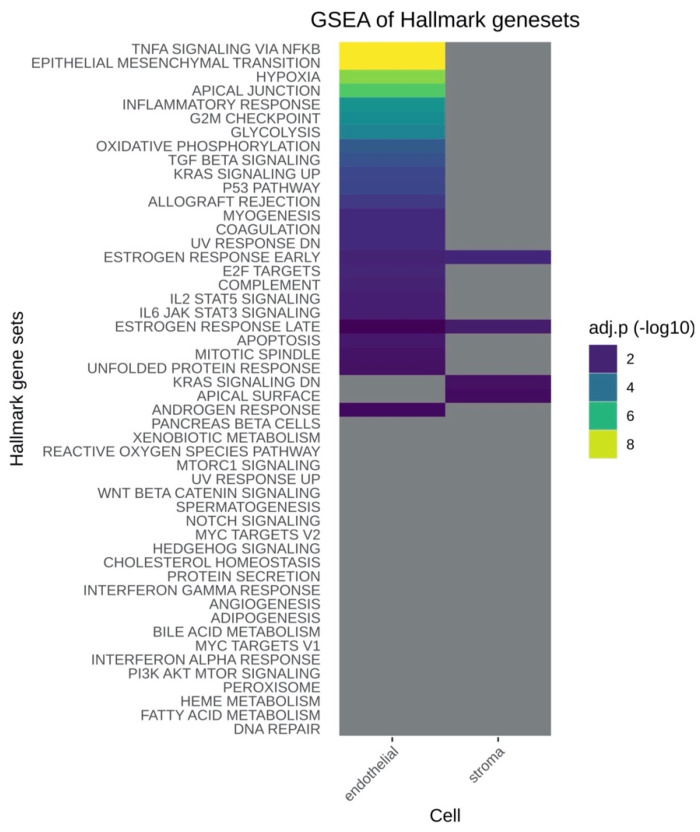
GSEA (Gene Set Enrichment Analysis) extracted from the next generation sequencing data of the stroma and Descemet membrane/endothelium (DmE) complex of donor corneas after 25 days of differential culture conditions in either 2% HPL or 2% FBS-containing medium (2%HPL vs 2%FBS). Grey clusters indicate no significant differences between 2%HPL and 2%FBS. Significant differential gene regulation is indicated by coloring the clusters according to adjusted *p*-values. Please also see Supplementary Data S4 for examples of the associated DGEA (Differential Gene Expression Analysis).

The GSEA analysis of hallmark gene sets showed differential expression patterns predominantly in the endothelial cells (Figure 4 and Supplementary Data S4). The most significant alterations were found in TNF-α signaling via NF-κB, epithelial mesenchymal transition and hypoxia (Supplementary Data S2). For TNF-α the most significant alterations in the associated DGEA were found as a 2.9 log2FC increase in 2%HPL of serpin family E member 1 or plasminogen activator inhibitor type 1 (PAI-1) and a 2.1 log2FC increase of PMEPA1. PAI-1 encodes a member of the serine proteinase inhibitor (serpin) superfamily (SERPINE1). This member is the principal inhibitor of tissue plasminogen activator (tPA) and urokinase (uPA), and hence is an inhibitor of fibrinolysis. PAI-1 can be upregulated by TNF-α signaling via NF-κB [29]. The molecular function has not yet been characterized for the corneal endothelium; however, it has been shown to stimulate human corneal epithelial cell adhesion, migration and epithelial corneal wound healing [30], and the related family member serine protease inhibitor A3K has been shown to protect the rabbit corneal endothelium from barrier function disruption induced by TNF-α [31]. SERPINE1 was upregulated in CEC and CSK in our dataset (Supplementary Data S1). PMEPA1 (Prostate Transmembrane Protein, Androgen Induced 1) encodes a transmembrane protein that contains a Smad interacting motif (SIM). Expression of this gene is induced by androgens and TGF-β, and the encoded protein suppresses the androgen receptor and TGF-β signaling pathways through interactions with Smad proteins. In the corneal endothelium, a PMEPA1 downregulation has been found in patients with Fuchs endothelial dystrophy, however, the exact molecular function is unclear [32].

Serpin family E member 1 and PMEPA1 are also among the three most significant gene alterations of the epithelial mesenchymal transition cluster. In addition, IGFBP3 (insulin like growth factor binding protein 3) is upregulated 2.5 log2FC in 2%HPL. The IGF family is composed of multiple ligands, receptors, and ligand binding proteins. The six IGF-binding proteins (IGFBPs) function primarily to sequester and inhibit IGF ligands. IGFBP-3 can inhibit IGF-1, thus the degree of fibrosis in the corneal stroma. In the corneal epithelium IGFBP-3 may function as a major stress response protein [33]. However, the specific function of IGFBP-3 in the corneal endothelium to date remains unknown.

In addition to SERPINE1 and IGFBP3, angiopoietin like 4 (ANGPTL4, 2.8 log2FC increase) and HMOX1 (DmE 3.44 log2FC, stroma 1.35 log2FC increase in 2% HPL) are relevant to the GSEA hypoxia cluster. They are discussed among the top 10 regulated genes (Supplementary Data S3).

GSEA analysis for the DmE apoptosis cluster (Supplementary Data S4) showed the highest significance values for the upregulation of GADD45B by 1.32 2log2FC, and once again of HMOX1 by 3.44 log2FC and biglycan by 1.38 log2FC. The molecular function of growth arrest- and DNA-damage-inducible 45β gene in the cornea to date is not clear. However, in RGCs, GADD45b was upregulated in response to oxidative stress, aging and elevated intraocular pressure and protected from cell death through oxidative stress, TNF-α cytotoxicity, and glutamate excitotoxicity in vitro as well as in a knockout mouse model [34].

Biglycan (BGN) is a small leucine-rich repeat proteoglycan (SLRP) which is found in a variety of extracellular matrix tissues. The effects of biglycan on CECs is unclear. Pinto et al. found a positive correlation between BGN and anti-apoptotic gene signatures (BCL2, BCL2L2, BCL2A1, IQSEC2, and BCL2L1) and an inverse correlation with pro-apoptotic gene signatures (CASP3, CASP6, CASP5, CASP8, CASP10, FASN, BAK, and BIK) in gastric cancer human samples [35]. However, IL-1-induced apoptosis of transformed rabbit corneal keratocytes was enhanced by biglycan [36].

In the GSEA hallmark TGF-β signaling analysis of the DmE (Supplementary Datas S2 and S4), in addition to the aforementioned upregulations of SERPINE1 and PMEPA1, LTBP2 (latent transforming growth factor beta binding protein 2, log2FC 1.35) and LEFTY2 (left-right determination factor 2) were significantly upregulated. We discuss LEFTY2 in the Top 10s (Supplementary Data S3).

GSEA analysis for the stroma showed significant alterations mainly in the early and late estrogen response, KRAS signaling and apical surface. For early estrogen response, the downregulations of ANXA9, FASN and RAPGEFL1 were highly significant. The ANXA9 downregulation are discussed among the top 10 regulated genes.

FASN (fatty acid synthase) encodes a multifunctional protein which works as an enzymatic system to catalyze fatty acid synthesis. High FASN expression has been found in several cancers such as breast and gastric cancer. FASN has been investigated as a possible chemotherapeutic target and FAS inhibitors are an active area in drug research [37]. Its expression or function in the cornea has not yet been described.

RAPGEFL1 (Rap guanine nucleotide exchange factor like 1) is a protein-coding gene involved in G protein-coupled receptor signaling pathways and guanyl-nucleotide exchange factor activity. It is expressed in multiple tissues. Its specific molecular functions are still unclear [38]. Its expression or function in the cornea has not yet been described.

For late estrogen response, the downregulations of CXCL14, ANXA9 and FABP5 in 2%HPL were highly significant. CXCL14 (C-X-C motif chemokine ligand 14) displays chemotactic activity for monocytes and activates them in the presence of the inflammatory mediator prostaglandin-E2 (PGE2). It is also a potent chemoattractant and activator of dendritic cells. It is implicated in homing in these cells, and can stimulate the migration of activated NK cells [39]. Interestingly, CXCL14 was downregulated in CEC and CSK in our dataset (Supplementary Data S1) and could lead to lower rejection rates after the transplantation of 2%HPL corneas. However, future trials are needed for clarification. FABP5 (fatty acid binding protein 5) encodes a cytoplasmatic protein that binds long-chain fatty acids and other hydrophobic ligands. Song et al. further examined the role of FABP5 in radiation-induced human skin fibrosis and found that an overexpression of FABP5 resulted in nuclear translocation of SMAD2 and significant activation of the profibrotic TGF-β signaling pathway in human fibroblasts [40]. FABP5’s profibrotic effect was also demonstrated in pulmonary artery fibrosis, where it was highly upregulated in primary pulmonary adventitial fibroblasts under TGF-β1 stimulation via the wnt/β-catenin pathway [41]. Its expression or function in the cornea has not yet been described. However, a profibrotic effect can be assumed.

For KRAS signaling, the upregulation of SELENOP and PTGFR in 2%HPL were highly significant. SELENOP (selenoprotein P) is considered an extracellular antioxidant and is involved in the transport of selenium to extrahepatic tissues via apolipoprotein E receptor-2 (apoER2) [42]. SELENOP is considered a key molecule in protecting the ocular surface cells against environmental oxidative stress. In a rat dry eye model, an improvement in the corneal dry eye index and the suppression of oxidative stress markers were observed in the SELENOP eye drop group [43]. PTGFR (prostaglandin F receptor) encodes a member of the G-protein coupled receptor family. In humans, PTGFR is expressed in the eye (ciliary muscle, choroid plexus, sclera, endothelium and smooth muscle cells of blood vessels of the iris). Animal and human studies have found PTGFR to be involved in modulating intraocular pressure [44]. Its expression or specific function in the cornea has not yet been described.

For the apical surface, the downregulations of MAL and GATA3 in 2%HPL were highly significant. MAL (mal, T cell differentiation protein) encodes a highly hydrophobic integral membrane protein belonging to the MAL family of proteolipids. It is localized to the endoplasmic reticulum of human T-cells where it acts as a linker protein in T-cell signal transduction [45]. Its expression or function in the cornea has not yet been described. GATA3 (GATA binding protein 3) encodes a protein which belongs to the GATA family of transcription factors. It acts as a regulator of T-cell development and is involved in the embryonic development of various cell types and tissues [46]. GATA3 is also important for inflammatory and humoral immune responses via regulation of the development of innate lymphoid cells (ILCs) and T helper cells. GATA3 stimulates the secretion of several interleukins (IL-4, IL-5, and IL-13) from Th2 cells in humans, promoting allergic responses [47]. In the eye, GATA3 expression has been detected in the posterior part of the lens [48]. Its expression or function in the cornea has not yet been described.

## 3. Discussion

In this paired culture study, we showed that 2%HPL is a suitable xeno-free supplement substitution for 2%FBS in human donor cornea organ culture. We detected no significant alterations in morphology of the CECs during the 25-day differential culture period. The ECL was significantly lower in 2%HPL vs. 2%FBS. NGS showed differences in gene expression predominantly in the DmE (1644 genes) and less in the stroma (217 genes). CSK markers were not influenced. Some CEC markers showed mild alterations. Overall, 2%HPL vs. 2%FBS led to the upregulation of cytoprotective, anti-inflammatory and anti-fibrotic genes (e.g., HMOX1, SERPINE1, ANGPTL4, LEFTY2, GADD45B, PLIN2, PTX3, GFRA1 and 2) and the downregulation of pro-inflammatory/apoptotic genes (e.g., CXCL14, SIK1B, PLK5, PPP2R3B, FABP5, MAL, GATA3). This could prove favorable for 2%HPL cornea organ culture and transplantation.

Recent years have seen groundbreaking advances in targeted cell therapy approaches as an alternative to corneal transplantation [49]. The Kinoshita group from Japan recently published their five-year follow-up of the first 11 patients treated with an injection of cultured human CEC for endothelial failure [3]. Normal corneal endothelial function was restored in 10 of the 11 eyes [3]. These results are very promising for the most important indication for corneal transplantation in the industrialized world, i.e., endothelial failure [7]. The transplantation of in vitro cultured human CEC allows the quality control of cells prior to a minimally invasive injection, and the expansion of CECs could enable the treatment of numerous patients from one donor, replacing the one-to-one limitation of traditional corneal transplantation [3]. Nevertheless, larger long-term studies first have to verify the safety and fate of the CEC being injected into the anterior chamber, particularly in comparison to established endothelial keratoplasty techniques that also have excellent outcomes, e.g., Descemet membrane endothelial keratoplasty (DMEK) [7,50,51].

In 1997 Pellegrini et al. cultivated and expanded human limbal stem cells (LSC) ex vivo and successfully transplanted the cell sheets onto the corneal surface of two LSC-deficient patients [52]. Favorable results using this treatment for epithelial corneal failure were also reported by Tsai et al. and Rama et al. [53,54]. The number of transplanted LSC expressing ΔNp63α can be measured ex vivo and was found to be important for long-term graft survival [54].

Despite the recent advances in ex vivo LSC and CEC expansion, mimicking the complex mechanical properties, the surface curvature, the stromal cytoarchitecure and the transparency of the human corneal stroma by tissue engineering has seen little success to date [55,56,57]. Consequently, today and in the intermediate future, there is still a high demand for donor corneas. With an estimated 12.7 million people waiting for a corneal transplantation, only 1 in 70 of the needs are covered worldwide [58].

As an advancement to closed systems during organ culture and cold-storage, recent studies have investigated the possibility of using dynamic bioreactor systems that enable the control of intraocular pressure, culture with an air-liquid interface, different media for the epithelium and endothelium, the assessment of transparency, swelling, and measuring of the trans-epithelial electrical-resistance (TEER) as an in vivo functionality readout [59,60]. These approaches have the potential to ameliorate the quality of corneal grafts and storage time in eye banks in the future.

In the search for a suitable substitution for FBS for human donor cornea organ culture, it is crucial to define the demands for such a substance. Human CECs in vivo are postmitotic with an extremely low proliferation rate. Thus, cell loss cannot be compensated by cell division but only by an increase in the size of the remaining neighboring endothelial cells. This inevitably leads to an irregular increase in the area of the CECs and to a loss of the typical hexagonality. Gradual deviation from hexagonality by, e.g., pleomorphism and polymegathism of CECs, are typical signs of an aged cornea [10]. Corneal transparency and the visual function of the eye are largely ensured by the relative corneal dehydration, which is preserved by various mechanisms localized largely in the corneal endothelium. The two main endothelial cell functions are the conservation of corneal dehydration by active, i.e., energy-dependent, pump mechanisms (Na+-K+-ATPase) and the physical barrier function (‘leaky barrier’) that characterizes fluid and electrolyte influx [61,62]. Consequently, the endothelium is the most metabolically active, most important, as well as the most vulnerable layer of the human cornea, and so a minimization of ECL is the goal.

In our study, the CECs only showed mild alterations at TP1 and TP2, and no significant differences in morphology between the 2%HPL and 2%FBS groups. We have previously shown that areas of denuded endothelium (ECFA) represent a significant predictor for donor cornea exclusion from transplantation during culture. However, we did not find a correlation between phenotypic alterations and ECL [10]. In this study we saw that CECs in both groups maintained the characteristic, hexagonal monolayer, indicating generally healthy cells throughout the culture.

CECs show a marked physiological density decrease from the rim to the center of the cornea. The central ECD decreases with age. Thus, it amounts to about 3500–4000 cells/mm^2^ in newborns and can decrease to levels of about 1500–2500 cells/mm^2^ in older adults [61]. At the start of the differential culture both our groups had comparable ECDs. After 25 days of differential culture the 2%HPL ECD was higher (2179 vs. 2113 cells /mm^2^), while ECL was lower (−0.7% vs.−3.8%). 

Currently, a minimum ECD of 2000 cells/mm^2^ is recommended in Germany and most of Europe for the release of elective grafts [10,61]. Central ECD is therefore one of the most important parameters in evaluation of donor corneas. Of the 62 eye banks included in the 2010 European Eye Bank Association Directory, 47 used organ culture, 9 used hypothermia, and 6 used both methods: overall, 70% of corneas were stored by organ culture. The most common organ culture medium is Eagle’s minimum essential medium (MEM) with 2% FBS, although up to 8% FBS is used by some eye banks [12]. ECL, particularly in the early days of cornea organ culture, reached up to 30% of the initial density [63]. The ECL of modern organ culture protocols is usually around 5 ±5% [10]. Cold-storage intervals from 4 to 21 days in Optisol-GS previously led to an average decrease in endothelial viability of 9.5% to 16% [64].

Overall, organ culture and cold storage showed comparable clinical outcomes in terms of ECL, visual acuity and graft survival for PK [65], DALK and DMEK surgeries [66,67]. In terms of ECL, studies do not clearly favor one method. For instance, in a prospective, randomized clinical evaluation with 12 cornea pairs, the difference in ECL 1 year after PK did not prove statistically significant (15% organ culture and 20% cold storage) [65]. The storage method did not influence the ECL 1 year after 84 DALKs (4  ±  16%) [67]. One year following DMEK surgery with 644 (76.6%) organ culture donors and 197 (23.4%) cold storage donors, ECL was slightly greater (*p* = 0.022) in organ culture (38.3  ±  0.8%) compared to cold storage (34.7  ±  1.4%) cases [68].

Interestingly, we found a differential regulation of genes in CEC during 2%HPL culture that have been associated with an anti-apoptotic potential. Among them are the upregulation of GDNF family receptor alpha 1 and 2, as well as heme oxygenase 1 (HMOX1) and the downregulation of salt inducible kinase 1B. These are all discussed in Supplementary Data S3. In addition, GSEA analysis for the DmE apoptosis cluster showed the highest significance for the upregulation of GADD45B by 1.32 2log2FC and HMOX1 by 3.44 log2FC. In the TNF-α GSEA analysis, a 2.9 log2FC increase of SERPINE1 in 2%HPL was detected. Interestingly, these genes, which are associated with anti-apoptotic effects, were also significantly upregulated in our stromal samples and are discussed in the GSEA section. However, their possible anti-apoptotic effects in CECs will have to be validated in the future.

HPL has proven a suitable supplement for CEC culture before [28]. Thieme et al. used 5% FBS and 0.02% HPL for the expansion of primary human CEC. With a colorimetric metabolic activity assay, they found comparable viabilities in both groups for incubation times up to 25 days [69]. Furthermore, they quartered human donor corneas and found that pieces incubated for 2 weeks in 0.1 mg/mL HPL (0.01%) in Biochrome I showed a 21 (±10) % ECL compared with 67 (±12) % ECL when cultivated in 2% FBS in Biochrome I. However, they did not investigate the associated genetic alterations, and quartering donor corneas was traumatic for the endothelium and therefore resulted in increased ECL. In addition, the HPL concentration used was far lower than in our setup, which could also be responsible for the relatively high ECL [69]. 

Similarly, Petsoglou et al. showed a positive effect of 5% HPL on primary CEC growth, and maintenance of their cellular characteristics compared to 5% FBS. However, they found a high variation in terms of growth factor content between different HPL sources that also had differential effects on cell proliferation and marker expression [28]. They used two sources of HPL, a commercially available product, PLTMax^®^ human platelet lysate (HPL-M) from Merck, USA; and HPL-R, a gift from the Australian Red Cross, Sydney. The HPL-R had been aliquoted and stored at −80 °C for 2 years before use. Both types of HPL were prepared from pooled donor platelet samples without heparin and were thawed with no more than two freeze–thaw cycles. They found that heparin impaired the positive effect of HPL on cell growth, HPL increased ZO-1 and CD166 but not Na+/K+-ATPase expression in primary human CEC. They concluded that HPL can be considered as a supplement to replace FBS in primary human CEC culture, but that a standard quality-control, monitoring storage time and growth factor content, may need to be established, which we absolutely agree with [28]. The HPL used in this study was also generated by pooling multiple donors to limit lot variation and did not contain or need heparin for use. However, we did not evaluate alterations during organ storage in functional parameters such as ZO-1 or Na+/K+-ATPase expression, which should be considered in the future. 

These and our study strongly encourage further studies to find the ideal concentration, culture period and reliable protocol for HPL human donor cornea organ culture.

One of the most dreaded complications post-keratoplasty is allogeneic graft rejection, which can kill the graft’s donor stromal and endothelial cells. Epithelial and stromal donor cell loss is generally of minor concern as the cells are usually substituted by recipient cells that migrate onto and into the graft [49]. Endothelial rejection, however, is a major concern, as postmitotic CECs cannot be substituted and lose their pump function when they die. The donor graft consequently swells, loses transparency and fails [49]. The eye’s “immune privilege” can limit the risk of rejection mainly through the absence of corneal vascularity, which hinders delivery of immune elements, the absence of corneal lymphatics, which prevents delivery of antigens to T cells in lymph nodes, the expression of FAS ligand in the anterior chamber, which can induce apoptosis of stimulated FAS+ T cells, and an unusually low expression of MHC antigens [70].

FBS has been shown to induce pro-inflammatory genes before [17,18,19,71]. Interestingly, we found the differential regulation of genes during 2%HPL culture that could exert anti-vascular, anti-fibroblastic and anti-inflammatory effects. Among them is the upregulation of Left-right determination factor 2 (LEFTY2) in the DmE. Its expression in the cornea has not yet been described. However, LEFTY2 alleviates hepatic stellate cell activation and liver fibrosis by inhibiting the TGF-β1/Smad3 pathway [72]. The previously mentioned HMOX1 and ANGPTL4 were upregulated in the DmE as well as in the stroma (Supplementary Data S2). Their potential anti-inflammatory, anti-vascular and immunosuppressive effects were discussed above and in Supplementary Data S3 [73,74,75,76]. PMEPA1 (Prostate Transmembrane Protein, Androgen Induced 1) was strongly upregulated in the 2%HPL DmE and suppresses the androgen receptor and TGF-β signaling pathways through interactions with Smad proteins [32]. CXCL14 was downregulated in 2%HPL CEC and CSK in our dataset. CXCL14 is a potent chemoattractant and activator of dendritic cells, and downregulation could reduce the risk of rejection in 2%HPL corneal transplants [77].

Further potentially anti-inflammatory effects could be exerted by the upregulation of PLIN2 and PTX3, as well as the downregulation of pro-inflammatory genes (e.g., FABP5, MAL and GATA3) we detected in our 2%HPL stromal samples.

Limitations of this study include the fact that the variability in quality, growth factor content and source can significantly influence the results not just for FBS but also for HPL [28], which can be attenuated for HPL by pooling donors. Even though the analysis of 15 corneal pairs by NGS yielded robust data, further studies ex and in vivo that separately inhibit/knock-out the genes/proteins we outlined are necessary to test their individual relevance for, e.g., corneal endothelial cell survival and inflammation.

Finally, future clinical studies will have to show, whether the described effect on ECL and the potentially beneficial alterations in gene expression in 2%HPL organ culture corneas also translate into the clinic with better keratoplasty outcomes, e.g., lower rejection rates.

In conclusion, 2%HPL is a suitable xeno-free substitution for 2%FBS in human cornea organ culture, inducing less ECL and potentially beneficial alterations in gene expression.

## 4. Material and Methods

### 4.1. Organ Culture and Cornea Processing

Human donor corneas were procured according to German and European regulations and stored in organ culture as described previously [9,10,78]. We collected 32 human corneas unsuitable for transplantation from 16 human donors (age 69.3 ± 15.7 years), 38.5 ± 17.1 h after death (Table 1). In brief, corneas were procured by corneoscleral disc excision within 72 h post-mortem and then stored for 3 days in 2% FBS-containing medium (2%FBS, origin Australia, gamma irradiated, CEP No. R1-CEP 2001-032-REV 01, Biochrom GmbH, Berlin, Germany) in minimal essential medium (MEM w/Earle´s Salts, w/L-Glutamine w/o Sodium Bicarbonate, w/o NEAA, Biowest, Nuaillé, France) supplemented with 0.5% penicillin/streptomycin and 0.5% amphotericin B (both Sigma-Aldrich, St. Louis, MO, USA), 0.357% HEPES (Carl Roth GmbH + Co. KG, Karlsruhe, Germany) and 0.285% sodium hydrogen carbonate (Merck KGaA, Darmstadt, Germany) [9,10,78]. 

Following 3 days of 2%FBS culture (time point TP1) donor corneas were then evaluated (Figure 1) and washed 3 times with sterile phosphate-buffered saline (PBS, 0.1 M, Merck KGaA). The culture was continued for an additional 25 days with one cornea of the pair cultured in 2%FBS and one in 2%HPL, which was identical in medium composition but contained 2% HPL (ELAREM^TM^ Ultimate-FDi, US origin, viral-inactivated, fibrinogen-depleted, no heparin required—GMP Grade—PL BioScience GmbH, Aachen, Germany) instead of 2% FBS.

Corneas were randomly allocated to either 2%FBS or 2%HPL. Following 25 days of differential culture (28 days total culture time, time point TP2) culture was stopped, and corneas were re-evaluated and processed for NGS. A quality management system (QMS) according to ISO 9001 was used to standardize, document, and control the entire banking process.

### 4.2. Endothelial Cell Density and Morphology Evaluation

At TP1 and TP2, central endothelial cell density (ECD) and morphology were assessed microscopically (Leica DM IL EQ inverted microscope, Leica Microsystems, Wetzlar, Germany) using the parameters polymegethism, pleomorphism, granulation, vacuolization, segmentation of cell membranes, Descemet folds, trypan blue-positive cells and endothelial cell-free areas, using an established classification by standardized scores between grades 0 and 3. A score of 0 represented a normal endothelial cell layer morphology with no pathologic alterations, while a score of 3 would indicate a vastly altered and diseased endothelium. All scores were assessed by a blinded experienced eye bank technician (SS) of the cornea bank, as previously published [10]. ECD was also assessed by the blinded experienced eye bank technician (SS) from digital photomicrographs of the corneas by manual counting in duplicate using the fixed frame/L method (Gundersen 1977) with an overlay on the computer screen, which was previously calibrated using a microscopic calibration scale [79].

### 4.3. Cornea Processing for NGS

Following 25 days of differential culture (28 days total culture time), TP2 ECD and cell morphology evaluation, 15 cornea pairs were washed three times with sterile phosphate-buffered saline (PBS, 0.1 M, Merck KGaA). Then, the Descemet membrane/endothelium complex (DmE) was stained with trypan blue (Vioron 0.5 mL, Fluron GmbH, Ulm, Germany) for 1 min and peeled with non-toothed forceps. The DmE was then transferred into a 1.5 mL collection tube (Micro tube 1.5 mL, Sarstedt AG & Co. KG, Nümbrecht, Germany) containing 350 μL buffer RLT (Rneasy Micro Kit, Qiagen, Hilden, Germany) on ice. Then, the epithelium was thoroughly scraped off with a hockey knife and once again washed three times with PBS. The central stromal button was then trephined (8.5 mm diameter) and cut into quarters. Each quarter was placed into a separate collection tube containing 350 μL RLT buffer on ice. DmE and stroma samples were disrupted and homogenized using a ball mill (Schwingmühle TissueLyser 2, Retsch GmbH, Haan, Germany) for 4 min at 25 Hz, and the lysate was centrifuged for 3 min at 14,000 rpm. The supernatant was transferred into 1.5 mL collection tubes and total RNA was extracted using the RNeasy Micro Kit (Qiagen) together with the RNase-free DNase Set (Qiagen) according to the manufacturer’s protocol. RNA quality was checked using the Bioanalyzer 2100 Total RNA Nano Assay (Agilent, Waldbronn, Germany) and the Quantus Fluorometer (Promega, Madison, WI, USA) was used to measure RNA quantity. RNA integrity number (RIN) values for the DmE samples ranged from 6.3 to 9.1 with a mean RIN of 7.5 and a mean RNA concentration of 65.9 ng/µL. RIN values for the stromal samples ranged from 4.3 to 8.0 with a mean RIN of 6.7 and a mean RNA concentration of 15.5 ng/µL. RIN values and RNA concentrations for all samples can be found in Supplementary Data S5. While the mean RIN value for stroma was below the industry standard of 7 or greater, the majority of the stromal RNA samples have low RIN values due to technical limitations of isolating RNA from corneal stroma. However, because the RIN values for the majority of the stroma samples are consistently lower, this facilitates reasonable comparison, yet we recognize it as a limitation in our study. Library preparation was performed with the Collibri 3′mRNA Library Preparation Kit (Invitrogen, Waltham, MA, USA) according to the manufacturer’s instructions. All samples were sequenced on an Illumina NextSeq 500 instrument (Illumina, San Diego, CA, USA) using 75 bp single-end mode. Sequencing yielded a mean coverage of approximately nine million reads per sample.

### 4.4. Next Generation Sequencing

FASTQ files were generated using bcl2fastq (Illumina). To facilitate reproducible analysis, samples were processed using the publicly available nf-core/RNA-seq pipeline version 3.5 [80] implemented in Nextflow 21.10.6 [81] using Docker 20.10.12 [82] with the minimal command. In brief, lane-level reads were trimmed using Trim Galore 0.6.7 [83] and aligned to the human genome (GRCh38.p13) using STAR 2.7.9a [84]. Transcript-level quantification was done by Salmon v1.5.2 [85]. All analysis was performed using custom scripts in R version 4.1.1 using the DESeq2 v.1.32.0 framework [86]. The 2%FBS group was set as the control, and so a log2 fold-change (log2FC) of 1 would indicate a gene expression twice as high in the 2%HPL compared to the 2%FBS samples.

### 4.5. Statistical Analyses

All data were expressed as mean ± standard deviation (SD). Statistical analyses were performed with SPSS version 22.0 (IBM, Chicago, IL, USA). Paired Student’s *t* tests were used to compare ECD and cell morphology parameter changes. A *p*-value ≤ 0.05 was considered statistically significant.

## Figures and Tables

**Table 1:** Gender, age, death to cornea collection time and cause of death of the cornea donors included in this study.

Donor	Gender	Age (Years)	Death to Cornea Collection Time (Hours)	Cause of Death
1	f	79	61.17	hemorrhagic shock
2	m	59	59.18	hemorrhagic shock; thoracoabdominal aneurysm of Aorta
3	m	61	38.17	multiple organ failure; liver cirrhosis
4	f	81	14.25	multiple organ failure; hepatic failure
5	m	64	71.87	multiple organ failure; liver cirrhosis
6	f	85	25.30	renal failure
7	f	84	37.48	respiratory failure; fall
8	f	95	63.33	basilar artery occlusion, ischemic stroke
9	f	31	23.65	pancreatic carcinoma
10	m	62	39.85	hemorrhagic shock; perforated coronary bypass
11	f	54	32.17	septic shock; acute myeloid leukemia
12	f	57	30.17	pancreatic carcinoma
13	m	73	41.68	cerebral hemorrhage
14	m	67	32.13	septic shock; carcinoma of colon
15	m	72	17.07	hepatic failure; bladder carcinoma
16	f	84	28.58	rectal carcinoma
Mean		69.25	38.50	
SD		15.72	17.08	

**Table 2:** Changes in endothelial cell density (ECD) of paired donor corneas at TP1 (after 3 days of preculture) and TP2 (after 25 days of differential culture in either 2%FBS or 2%HPL).

Donor	Culture	ECD TP1 2%HPL Cells/mm^2^	ECD TP2 2%HPL Cells/mm^2^	ECD Change Cells/mm^2^	ECD Change%	Culture	ECD TP1 2%FBS Cells/mm^2^	ECD TP2 2%FBS Cells/mm^2^	ECD Change Cells/mm^2^	ECD Change %
1	HPL	1935	1995	60	3.10	FBS	1905	2040	135	7.09
2	HPL	2050	2055	5	0.24	FBS	2035	1950	−85	−4.18
3	HPL	2600	2560	−40	−1.54	FBS	2375	2295	−80	−3.37
4	HPL	2630	2590	−40	−1.52	FBS	2535	2515	−20	−0.79
5	HPL	1970	1940	−30	−1.52	FBS	1855	1840	−15	−0.81
6	HPL	2485	2440	−45	−1.81	FBS	2520	2465	−55	−2.18
7	HPL	1610	1570	−40	−2.48	FBS	1350	1310	−40	−2.96
8	HPL	1885	1870	−15	−0.80	FBS	1825	1750	−75	−4.11
9	HPL	2094	2088	−6	−0.29	FBS	2340	2125	−215	−9.19
10	HPL	2610	2525	−85	−3.26	FBS	2720	2560	−160	−5.88
11	HPL	2315	2400	85	3.67	FBS	2445	2255	−190	−7.77
12	HPL	2220	2125	−95	−4.28	FBS	2300	2230	−70	−3.04
13	HPL	2095	2105	10	0.48	FBS	2350	2200	−150	−6.38
14	HPL	2375	2325	−50	−2.11	FBS	2490	2285	−205	−8.23
15	HPL	2290	2335	45	1.97	FBS	2255	2255	0	0.00
16	HPL	1945	1935	−10	−0.51	FBS	1910	1725	−185	−9.69
MEAN		2194.31	2178.63	−15.69	−0.67		2200.63	2112.50	−88.13	−3.84
SD		297.35	288.14	48.90	2.17		355.47	330.99	93.11	4.21

**Table 3:** Evaluation and scoring of endothelial cell layer alterations after 3 days of organ culture in 2% FBS medium (2%FBS; time point 1) followed by 25 days of differential culture in either 2%FBS or 2% HPL containing medium (2%HPL; time point 2) using phase contrast microscopy.

	Time Point		Polymegethism	Pleomorphism	Granulation	Vacuolization	Segmentation of Cell Membranes	Endothelial Cell-Free Areas	Descemet Folds	Trypan Blue-Positive Cells
2% HPL	1	Mean	1.44	1.19	0.00	0.13	0.94	0.13	1.88	0.00
		SD	0.73	0.40	0.00	0.34	0.25	0.50	0.50	0.00
2% HPL	2	Mean	1.38	1.25	0.00	0.06	1.00	0.00	2.13	0.00
		SD	0.62	0.45	0.00	0.25	0.00	0.00	0.50	0.00
2% FBS	1	Mean	1.38	1.44	0.00	0.06	1.00	0.00	1.94	0.00
		SD	0.72	0.63	0.00	0.25	0.00	0.00	0.57	0.00
2% FBS	2	Mean	1.50	1.31	0.00	0.13	0.94	0.00	2.06	0.00
		SD	0.63	0.60	0.00	0.34	0.25	0.00	0.44	0.00

All scores (0–3) were assessed by a blinded experienced eye bank technician. A score of 0 represents a normal endothelial cell layer morphology with no pathologic alterations, while a score of 3 would indicate a vastly altered and diseased endothelium.

**Table 4:** Top 10 differentially up- and downregulated genes of the corneal endothelium and stroma following 25 days of paired culture in either 2%FBS or 2%HPL.

		Gene	Full Name	Molecular Function	Mean	log2FC
E N D O U P	1	GFRA1	GDNF family receptor alpha 1	glial cell-derived neurotrophic factor receptor activity	10.67	4.46
	2	GFRA2	GDNF family receptor alpha 2	glial cell-derived neurotrophic factor receptor activity	9.80	4.07
	3	CST1	cystatin SN	cysteine-type endopeptidase inhibitor activity	50.55	3.97
	4	S1PR5	sphingosine-1-phosphate receptor 5	G protein-coupled receptor activity	23.90	3.89
	5	C5orf46	chromosome 5 open reading frame 46	unclear	46.64	3.86
	6	DCX	doublecortin	microtubule binding, protein kinase binding	11.45	3.81
	7	LEFTY2	left-right determination factor 2	cytokine activity	50.76	3.74
	8	FNDC1	fibronectin type III domain containing 1	activator of G protein signaling	159.43	3.67
	9	NEURL1B	neuralized E3 ubiquitin protein ligase 1B	ubiquitin protein ligase activity	28.72	3.45
	10	HMOX1	heme oxygenase 1	protein homodimerization activity, oxidoreductase activity	849.53	3.44
E N D O D O W N	1	CACNA1F	calcium voltage-gated channel subunit alpha1 F	ion channel activity, high voltage-gated calcium channel activity	5.11	−2.90
	2	SIK1B	salt inducible kinase 1B	transferase activity, transferring phosphorus-containing groups, protein tyrosine kinase activity	8.18	−2.89
	3	TTC23L	tetratricopeptide repeat domain 23 like	unclear	8.26	−2.76
	4	CCDC201	coiled-coil domain containing 201	unclear	5.13	−2.70
	5	LCN12	lipocalin 12	transporter activity, retinoic acid binding	12.63	−2.54
	6	GALNT16	polypeptide N-acetylgalactosaminyltransferase 16	carbohydrate binding, polypeptide N-acetylgalactosaminyltransferase activity	5.55	−2.52
	7	RANBP3L	RAN binding protein 3 like	SMAD binding activity	118.52	−2.49
	8	B3GALT1	Beta-1,3-galactosyltransferase 1	galactosyltransferase activity, UDP-galactose:beta-N-acetylglucosamine beta-1,3-galactosyltransferase activity	7.00	−2.48
	9	DCC	DCC netrin 1 receptor	transmembrane signaling receptor activity, axon guidance	89.05	−2.45
	10	ETNPPL	polo like kinase 5	regulatory kinase of cell cycle, apoptosis	567.33	−2.45
S T R O M A U P	1	MKX	mohawk homeobox	sequence-specific DNA binding, DNA-binding transcription activator activity, RNA polymerase II-specific	7.36	1.83
	2	ANGPTL4	angiopoietin like 4	enzyme inhibitor activity (inactivation of the lipoprotein lipase LPL)	156.66	1.68
	3	HMOX1	heme oxygenase 1	protein homodimerization activity, oxidoreductase activity	428.25	1.35
	4	PLIN2	perilipin 2	lipid globule surface membrane material	2202.47	1.29
	5	PDK4	pyruvate dehydrogenase kinase 4	protein kinase activity	106.98	1.26
	6	TFPI2	tissue factor pathway inhibitor 2	serine-type endopeptidase inhibitor activity, peptidase inhibitor activity	78.66	1.25
	7	PTX3	pentraxin 3	virion binding, (1->3)-beta-D-glucan binding	705.56	1.24
	8	ESM1	endothelial cell specific molecule 1	integrin binding, hepatocyte growth factor receptor binding	50.20	1.12
	9	LPXN	leupaxin	focal adhesion protein, cell type-specific signaling	58.77	1.07
	10	CBL	Cbl proto-oncogene	cell signaling and protein ubiquitination	239.57	1.06
S T R O M A D O W N	1	ANXA8L1	annexin A8 like 1	calcium ion binding, calcium-dependent phospholipid binding	48.27	−8.37
	2	PPP2R3B	protein phosphatase 2 regulatory subunit B’’beta	calcium ion binding, protein serine/threonine phosphatase activity	19.56	−4.37
	3	SLURP1	secreted LY6/PLAUR domain containing 1	cytokine activity	31.10	−3.93
	4	GJA4	gap junction protein alpha 4	gap junction channel activity	11.78	−3.42
	5	LYPD2	LY6/PLAUR domain containing 2	post-translational modification: synthesis of GPI-anchored proteins	15.07	−3.33
	6	CEACAM7	CEA cell adhesion molecule 7	Post-translational modification: synthesis of GPI-anchored proteins	8.64	−3.17
	7	FSTL4	follistatin like 4	calcium ion binding	7.43	−2.90
	8	PIGR	polymeric immunoglobulin receptor	immunoglobulin receptor activity (innate immune system)	27.42	−2.81
	9	ANXA9	annexin A9	calcium ion binding, phospholipid binding	15.02	−2.80
	10	PCDH19	protocadherin 19	calcium-dependent cell-adhesion protein	42.01	−2.51

**Table 5:** Expression levels of known marker genes for stromal and corneal endothelial cells following differential organ culture in either 2%HPL or 2%FBS for 25 days.

Gene_Name	baseMean	log2FoldChange	padj	sig
Stroma				
LUM	7339.60	−0.04	0.97	Non-sig.
ALDH3A1	5061.16	−0.20	0.81	Non-sig.
ALDH1A1	360.23	−0.04	0.96	Non-sig.
COL3A1	322.41	−0.63	0.62	Non-sig.
KERA	247.90	−0.43	0.56	Non-sig.
THY1	119.26	−0.06	0.96	Non-sig.
ACTA2	92.73	0.40	0.64	Non-sig.
TNC	52.40	0.16	0.91	Non-sig.
Endothelium				
PTGDS	7862.98	−1.27	0.0000	Sig. genes
AQP1	4592.68	−0.26	0.3667	Non-sig.
ATP1A1	2506.61	−0.69	0.0000	Sig. genes
SLC4A11	2153.22	−1.24	0.0000	Sig. genes
COL8A2	1929.74	−0.05	0.8879	Non-sig.
CDH2	1667.72	0.69	0.0000	Sig. genes
SLC4A4	1644.20	−0.47	0.0406	Sig. genes
TJP1	579.02	−0.27	0.3754	Non-sig.
ENO2	578.72	0.10	0.7347	Non-sig.
VDAC2	532.68	−0.14	0.1763	Non-sig.
VDAC3	522.33	−0.25	0.0629	Non-sig.
CLCN3	475.47	0.16	0.5404	Non-sig.
ACTA2	178.71	0.90	0.1877	Non-sig.
F11R	100.05	−0.19	0.6834	Non-sig.
CLCN2	9.39	−0.29	0.7173	Non-sig.

## Data Availability

Data and materials are available on request.

## Supplementary Materials

The following supporting information can be downloaded at: https://www.mdpi.com/article/10.3390/ijms24032882/s1. References [87,88,89,90,91,92,93,94,95,96,97,98,99,100,101,102,103,104,105,106,107,108,109,110,111,112,113,114,115,116,117,118,119,120,121,122,123,124,125,126,127,128,129,130,131,132,133,134,135,136] are cited in the supplementary materials.
