# Supplementary material for: Cytoprotective Effects of Human Platelet Lysate during the Xeno-Free Culture of Human Donor Corneas
Source: Int J Mol Sci. 2023 Feb 2;24(3):2882. doi: 10.3390/ijms24032882 (PMC9917909; doi:10.3390/ijms24032882)
Supplement: Supplementary file 1 [file ijms-24-02882-s001.zip › supplementary Data S3 .pdf]

Suppl. Data S3: Word document characterizing the top 10 up and downregulated endothelial and stromal genes from Table 4 with focus on their relevance to the cornea and organ culture.

2.3.1. Top 10 DmE upregulation

In the DmE 2%HPL led to the upregulation of glial cell-derived neurotrophic factor (GDNF) family receptors. Neurotrophic factors, such as GDNF, NGF or BDNF have been shown to play a vital role in maintaining corneal epithelial stem cells in the limbus [1], and GDNF stimulated the proliferative activity of corneal epithelial cells as well as CSKs and exhibited anti-apoptotic activity [2].

The CST1 gene encodes cystatin SN, a component of human saliva, tears and urine, that is thought to play a protective role by inhibiting cysteine proteases. The conjunctiva is the main producer of cystatin SN in the anterior eye [3].

The importance of cytstatin SN for CECs has not been investigated yet, however, CECs have been shown to secrete proteinase and proteinase inhibitors and an imbalance is suspected to contribute to e.g. the irregular thickening of the Descemet membrane seen in FECD [4].

Sphingosine-1-phosphate receptor 5 encodes a G protein-coupled receptor which binds the lipid signaling molecule sphingosine 1-phosphate (S1P). Sphingosine 1-phosphate (S1P) is a pleiotropic lysophospholipid mediator involved in many cellular responses, including transient calcium mobilization, activation of MAP kinase signaling, inhibition of adenylyl cyclase and increased cell migration. Sphingosine 1-phosphate promotes endothelial cell barrier integrity by Edg-dependent cytoskeletal rearrangement in endothelial vascular cells [5]. The effect of S1P on CECs to date is still unknown.

C5orf46 is also known as sssp1, or skin and saliva secreted protein 1. Differential expression of this gene has been shown for the corneal epithelium, however, its molecular function and the relevance for CECs remain unclear [6].

Doublecortin (DCX) is a microtubule-associated protein expressed by neuronal precursor cells and immature neurons in embryonic and adult cortical structures.

Due to the nearly exclusive expression of DCX in developing neurons, this protein has been used increasingly as a marker for neurogenesis. In the cornea, a member of this family, doublecortin domain containing 5, so far has only been detected in human limbal epithelial stem and mature cells with an unknown.[7]

Left-right determination factor 2 (LEFTY2) encodes a member of the TGF-β family of proteins. The encoded protein is secreted and plays a role in left-right asymmetry determination of organ systems during development [8]. Its expression in the cornea has not been described yet. However, LEFTY2 alleviates hepatic stellate cell activation and liver fibrosis by inhibiting the TGF-β1/Smad3 pathway, which could also be beneficial to CECs [8].

FNDC1 (fibronectin type III domain-containing protein 1), also known as AGS8, is an activator of G protein signaling and involved in the cellular response to hypoxia, the apoptotic process of cardiac muscle cells and protein phosphorylation [9]. However, the specific function of the gene in the cornea is unknown.

NEURL1B (neuralized E3 ubiquitin protein ligase 1B) enables ubiquitin protein ligase activity and is therefore involved in ubiquitin-dependent endocytosis. It has been shown to be involved in the downregulation of the notch pathway through influencing the stability and activity of several notch ligands. NEURL1B is downregulated in keratoconus compared to non-keratoconus corneas, however the specific effect of this gene in keratoconus is unknown [10]. The importance of NEURL1B in CECs is also still unknown.

HMOX1 (heme oxygenase 1), a member of the heat shock protein family, cleaves heme to form biliverdin in heme catabolism. HMOX1 plays a key role as a sensor and regulator of cellular oxidative stresses and exhibits cytoprotective effects by catabolizing free heme and preventing it from sensitizing cells to undergo apoptosis [11]. Regarding its function in the cornea, it was found that hyperosmotic stress increases the mRNA expression and protein production of HMOX1 in human corneal epithelial cells, potentially exerting a cytoprotective effect [12]. Its function in CECs is still unknown. Interestingly, in our dataset HMOX1 was upregulated in the DmE as well as the stroma (Suppl. Data 1).

2.3.2. Top 10 DmE downregulation

CACNA1F (calcium voltage-gated channel subunit alpha1 F) encodes a multipass transmembrane protein that mediates the influx of calcium ions into the cell. In the eye, mutations of this gene can cause X-linked diseases, including congenital stationary night blindness, Aland-Island eye disease (also known as Forsius-Eriksson type ocular albinism) and cone-rod dystropia [13]. However, the specific function of the gene in CECs is still unknown.

SIK1B (salt inducible kinase 1B) encodes a serine/threonine protein kinase a member of the adenosine monophosphate-activated kinase (AMPK) family. Its catalytic activity is required for p53 phosphorylation in response to loss of adhesion and therefore induces p53-dependent apoptosis. Downregulation of SIK1 compromises p53 function and permits cells to grow independently of their anchoring [14]. The specific effects of SIK1B in CECs are unknown. However, an overall antiapoptotic effect with lower expression can be assumed.

TTC23L (tetratricopeptide repeat domain 23 like) is a protein coding gene located on chromosome 5, that is predicted to be located in cytoplasm, microtubule cytoskeleton, and midbody [15]. Its expression in the cornea and molecular mechanism has not been described yet.

CCDC201 (coiled-coil domain containing 201) is a protein coding gene located on chromosome 7 [15]. Its expression in the cornea and molecular mechanism has not been described yet.

LCN12 (lipocalin 12) is a protein coding gene located on chromosome 9. Members of the lipocalin family bind to specific cell-surface receptors of small hydrophobic ligands. They exhibit roles in retinol transport, olfaction, pheromone transport, and prostaglandin synthesis [16]. In a large-scale genome-wide association study of keratoconus including 4,669 cases and 116,547 controls the authors identified a significant association with 36 genomic loci, LCN12 being a member of one of these regions [17]. However, Its molecular function in CECs has not been described yet.

GALNT16 (polypeptide N-acetylgalactosaminyltransferase 16) encodes a protein, which works as a polypeptide transferase. Changes in GALNT expression, and therefore alterations in GalNAc O-linked glycosylation, may directly influence molecules implicated in aspects of epithelial-mesenchymal transition (EMT) [18]. The importance in CECs to date is not clear.

RANBP3L (RAN binding protein 3 like) is involved in the TGF-β/BMP signaling pathway where it regulates the nuclear export of Smad proteins. It plays an important role for mesenchymal cell differentiation in bone development, negative regulation of osteoblast differentiation, and positive regulation of myoblast differentiation.

The expression of RANBP3L in human CECs has previously been described, however its molecular function remains unclear [19].

B3GALT1 (beta-1,3-galactosyltransferase 1) encodes a type II membrane-bound glycoprotein that transfers galactose from UDP-alpha-D-galactose to substrates with a terminal beta-N-acetylglucosamine (beta-GlcNAc) residue. To date expression of this gene has only been found in the colon and brain [20]. Its function in the cornea has not been described yet.

DCC (deleted in colorectal cancer netrin 1 receptor) encodes a netrin 1 receptor, which is a transmembrane protein and member of the immunoglobulin superfamily of cell adhesion molecules. When bound to Netrin-1, DCC activates downstream signaling partners such as MAP kinase, focal adhesion kinase (FAK) or Src kinases [21]. In the eye, DCC is broadly expressed in the developing retina by retinal ganglion cells (RGC), where it is essential for RGC intraretinal axon guidance [22].

Netrin 1 simultaneously suppresses corneal inflammation and neovascularization in the cornea, however the DCC receptor was not responsible and detectable in these experiments [23]. Its molecular function in CECs remains unclear.

Polo-like kinases (PLKs) are regulatory serine/threonin kinases of the cell cycle involved in mitotic entry, mitotic exit, spindle formation, cytokinesis, and meiosis. Mouse PLK5 is a DNA damage inducible gene. Ectopic expression of PLK5 leads to cell cycle arrest in G1, decreased DNA synthesis, and apoptosis, a characteristic it shares with PLK3 [24]. The 2% HPL downregulation of PLK5 in the DmE could be one of the factors promoting CEC survival.

2.3.3. Top 10 stroma upregulation

MKX (mohawk homeobox), also known as iroquois homeobox protein-like 1 encodes an iroquois (IRX) family-related homeobox protein that is mainly known for stimulating tendon differentiation during embryological development. The amount of soluble collagens, mRNA levels of *Col1a1* and *Col1a2*, and decorin, a proteoglycan regulating collagen fiber formation, were decreased in tendons of MKX null mice [25]. As the corneal stroma also mainly consists of collagen I and proteoglycans [26], MKX could play an important role in corneal stroma ECM homeostasis, however, this has not been investigated so far.

ANGPTL4 is induced under hypoxic conditions in various cell types and is the target of peroxisome proliferator-activated receptors. The encoded protein is directly involved in regulating lipid metabolism and vascular biology. Corneal vascular endothelial growth factor-induced in vivo angiogenesis and vascular leakiness were significantly inhibited by the addition of ANGPTL4 [27].

Ex vivo, ANGPTL4 was able to reverse the fibroblast-to-myofibroblast differentiation promoting wound repair with minimizing scar formation in dermal fibroblasts [28]. Interestingly, ANGPTL4 was upregulated in CECs and CSKs in our dataset (Suppl. Data 1) and could potentially suppress corneal vascularization and scarring in 2%HPL cultured corneas.

HMOX1 (heme oxygenase 1), is also found among the top 10 upregulated genes in the DmE and was previously described. The molecular

function in CSK is unclear. Nevertheless, HMOX-1 has been shown to promote the immunosuppressive properties of rat and human MSCs, which could lead to lower rejection rates after the transplantation of 2%HPL corneas [29].

PLIN2 (perilipin 2) encodes a protein also known as adipose differentiation-related protein, which belongs to the perilipin family and is involved in intracellular lipid storage. PLIN2, is known to be transcriptionally activated by peroxisome proliferator-activated receptor (PPAR) signaling. Previous studies showed that PPAR-γ activation can also suppress TGF-β1-induced fibrogenesis in various organs and tissues by cell/tissue specific mechanisms, among them the inhibition of Myo-SF induction in the cornea [30]. Whether PLIN2 induction through 2%HPL in our stromal samples coincides with a potential antifibrotic effect through PPAR-γ activation is unclear.

PDK4 (pyruvate dehydrogenase kinase 4) encodes a mitochondrial protein with a histidine kinase domain. Expression of this gene is regulated by insulin, retinoic acid and glucocorticoids. PDK4 helps to decrease metabolism and conserve glucose by decreasing its conversion to acetyl-CoA. In the human cornea PDK4 has been shown to be highly expressed by CSK. The exact molecular function to date is not clear [31].

TFPI2 (tissue factor pathway inhibitor 2) encodes a member of the Kunitz-type serine proteinase inhibitor family. The protein inhibits several serine proteases including plasmin, factor Xa, factor VIIa/tissue factor, trypsin, chymotryspin and plasma kallikrein. In the eye TFPI2 stimulates proliferation of retinal pigment epithelial cells, but not the growth of fibroblasts or vascular endothelial cells in vitro [32]. Whether TFPI2 also stimulates the proliferation of CSK remains unclear.

PTX3 (pentraxin 3) encodes pentraxin-related protein PTX3, also known as TNF-inducible gene 14 protein, which is a member of the pentraxin protein family. In response to inflammatory stimuli such as IL-1 and TNF-α, PTX3 is induced in multiple mesenchymal and epithelial cell types [33]. PTX3s anti-angiogenic effect is predicted to play a protective role on pathological angiogenesis in diabetic retinopathy [34].

HC-HA/PTX3 (a unique matrix consisting of high molecular weight hyaluronic acid (HA) covalently linked with heavy chain 1 (HC1) from inter-α-trypsin and further complexed with PTX3 reverted human corneal fibroblasts and myofibroblasts to keratocytes by activating BMP (bone morphogenetic protein) signaling [35]. This antifibrotic and anti-inflammatory effect has also been proven in lung fibrosis and could be beneficial to 2%HPL donor corneas [36].

ESM1 (endothelial cell specific molecule 1) encodes a secreted protein also known as endocan, which is mainly expressed in endothelial cells in the human lung and kidney but also in human CECs **[37]**. The molecular function of ESM1 in CSK to date is unknown.

Leupaxin is preferentially expressed in hematopoietic cells and is most homologous to the focal adhesion protein, paxillin. It may function in cell type-specific signaling by associating with PYK2, a member of the focal adhesion kinase family. The molecular function in the corneal stroma is still unclear [38].

Cbl (named after Casitas B-lineage Lymphoma) is a mammalian gene encoding the protein CBL which is an E3 ubiquitin-protein ligase involved in cell signaling and protein ubiquitination. Knockdown and inhibition of c-Cbl in the corneal epithelium decreased ligand-dependent ubiquitylation of the EGFR and prolonged receptor activity improving corneal epithelial wound

healing in vitro and in vivo [39]. The molecular function in the corneal stroma is still unclear.

2.3.4. Top 10 stroma downregulation

ANXA8L1 (Annexin A8 Like 1) encodes a member of the annexin family of evolutionarily conserved Ca2+ and phospholipid binding proteins. Atmospheric pressure cold plasma (APCP), a novel tool for tissue disinfection in medicine through induced reactive oxygen species (ROS), increased the expression of annexins (ANXA8L1 by 24.73 fold) in ex vivo human corneas most likely as a stress response [40]. The downregulation of ANXA8L1 in CSK could therefore be a sign of reduced stress in 2%HPL vs. 2%FBS culture.

PPP2R3B (protein phosphatase 2 regulatory subunit B) encodes a regulatory subunit of the calcium-dependent Ser/Thr phosphatase 2 (formerly named type 2A, PP2A). This phosphatase is implicated in negative regulation of G1/S transition of mitotic cell cycle and negative regulation of cell proliferation [41]. Diseases associated with PPP2R3B include Alzheimer Disease and it is discussed as a therapeutic target in inflammation and neurodegeneration [42]. PPP2R3 expression was found in the mouse cornea [43], its specific molecular function in CSK is still unknown.

SLURP1 (secreted LY6/PLAUR domain containing 1) encodes a member of the Ly6/uPAR family but lacks a GPI-anchoring signal sequence. Overexpression of SLURP1 in human corneal limbal epithelial (HCLE) cells stabilizes cell junctions and suppresses TNF-α-induced upregulation of pro-inflammatory cytokines IL-8, IL-1b, CXCL1 and CXCL2)[44]. The molecular function of SLURP1 in the corneal stroma remains unclear.

GJA4 (gap junction protein alpha 4) is also known as connexin-37 or Cx37, which is a member of the connexin gene family. Like other connexin proteins, it forms connections between cells known as gap junctions. Connexin-37 was shown to be present in the central rat cornea, yet its specific role for CSK remains unknown [45].

LYPD2 (LY6/PLAUR domain containing 2) is mainly expressed in esophagus, stomach and skin. Among its related functions are synthesis of GPI-anchored proteins, metabolism of proteins and post-translational modification [46].

In the cornea LYPD2 differential gene expression analysis revealed high expression in the limbal superficial epithelium but low expression in the corneal epithelium [47]. Its specific effect in CSKs is still unknown.

CEACAM7 (CEA cell adhesion molecule 7, also referred to as CGM2) encodes a cell surface glycoprotein and member of the CEA protein family. In the eye, CEACAM7 is expressed in conjunctival cells [48]. Its importance for CSK is still unknown.

FSTL4 (follistatin like 4) encodes a protein, that acts as a receptor for the brain-derived neurotrophic factor (BDNF) and shows calcium ion binding activity.

In the eye FSTL4 was found to be expressed in retinal ganglion cells [49]. Neurotrophic factors such as NGF or BDNF are important for corneal stromal homeostasis and wound healing. They are produced and released by stromal nerves, CSK and SF [50]. The exact molecular function of FSTL4 in CSK is still unclear.

PIGR (polymeric immunoglobulin receptor) encodes a poly-Ig receptor and member of the immunoglobulin superfamily. Overexpression of PIGR promotes cell transformation and tumor growth. Its pro-oncogenic function has been proven in pancreatic cancer and hepatocellular carcinoma [51].

Zhang et al. further showed that PIGR mRNA was upregulated in hepatic stellate cells in advanced liver fibrosis.[52] Its expression in the cornea has not been described yet.

ANXA9 (annexin A9), also known as pemphaxin or annexin 31, encodes a member of the annexin family, which are calcium-dependent phospholipid-binding proteins [53]. Zhou et al. predicted that ANXA9 mediated cell growth and migration through TGF-β signal transduction pathways [54]. Its expression in the cornea has not been described yet. However, as we mentioned before annexins are generally upregulated during cellular stress [55]. The downregulation of ANXA9 in CSK could therefore be a sign of less stress in 2%HPL vs. 2%FBS culture.

The protein encoded by protocadherin 19 (PCDH19) is a member of the delta-2 protocadherin subclass of the cadherin superfamily. The encoded protein is thought to be a calcium-dependent cell-adhesion protein that is primarily expressed in the brain. The protocadherin gene was transcriptionally active in the maturing P10 mouse cornea. The importance in the adult corneal stroma is unknown [56].

1. Qi, H.; Chuang, E.Y.; Yoon, K.C.; de Paiva, C.S.; Shine, H.D.; Jones, D.B.; Pflugfelder, S.C.; Li, D.Q. Patterned expression of neurotrophic factors and receptors in human limbal and corneal regions. *Mol Vis* **2007**, *13*, 1934-1941.
2. Gavrilova, N.A.; Borzenok, S.A.; Revishchin, A.V.; Tishchenko, O.E.; Ostrovkiy, D.S.; Bobrova, M.M.; Safonova, L.A.; Efimov, A.E.; Agapova, O.I.; Agammedov, M.B.; et al. The effect of biodegradable silk fibroin-based scaffolds containing glial cell line-derived neurotrophic factor (GDNF) on the corneal regeneration process. *Int J Biol Macromol* **2021**, *185*, 264-276, doi:10.1016/j.ijbiomac.2021.06.040.
3. De Roo, A.-K.; Foets, B.; van den Oord, J.J. Superficial Conjunctival Epithelium as the Main Producer of Protective Tear Component Cystatin SN. *Investigative Ophthalmology & Visual Science* **2014**, *55*, 1846-1846.
4. Theriault, M.; Parent, N.; Gendron, S.; Brunette, I.; Rochette, P.J.; Proulx, S. Secreted protease imbalance in Fuchs Corneal Endothelial Dystrophy. *Investigative Ophthalmology & Visual Science* **2018**, *59*, 1358-1358.
5. Garcia, J.G.; Liu F Fau - Verin, A.D.; Verin Ad Fau - Birukova, A.; Birukova A Fau - Dechert, M.A.; Dechert Ma Fau - Gerthoffer, W.T.; Gerthoffer Wt Fau - Bamberg, J.R.; Bamberg Jr Fau - English, D.; English, D. Sphingosine 1-phosphate promotes endothelial cell barrier integrity by Edg-dependent cytoskeletal rearrangement.
6. Kowtharapu, B.S.; Prakasam, R.K.; Murín, R.; Koczan, D.; Stahnke, T.; Wree, A.; Jünemann, A.G.M.; Stachs, O. Role of Bone Morphogenetic Protein 7 (BMP7) in the Modulation of Corneal Stromal and Epithelial Cell Functions. *Int J Mol Sci* **2018**, *19*, 1415, doi:10.3390/ijms19051415.
7. Albert, R.; Veréb Z Fau - Csomós, K.; Csomós K Fau - Moe, M.C.; Moe Mc Fau - Johnsen, E.O.; Johnsen Eo Fau - Olstad, O.K.; Olstad Ok Fau - Nicolaissen, B.; Nicolaissen B Fau - Rajnavölgyi, E.; Rajnavölgyi E Fau - Fésüs, L.; Fésüs L Fau - Berta, A.; Berta A Fau - Petrovski, G.; et al. Cultivation and characterization of cornea limbal epithelial stem cells on lens capsule in animal material-free medium.
8. Yang, Y.R.; Bu, F.T.; Yang, Y.; Li, H.; Huang, C.; Meng, X.M.; Zhang, L.; Lv, X.W.; Li, J. LEFTY2 alleviates hepatic stellate cell activation and liver fibrosis by regulating the TGF-β1/Smad3 pathway.

9. Anderegg, U.; Breitschwerdt, K.; Köhler, M.J.; Sticherling, M.; Haustein, U.F.; Simon, J.C.; Saalbach, A. MEL4B3, a novel mRNA is induced in skin tumors and regulated by TGF-beta and pro-inflammatory cytokines. *Exp Dermatol* **2005**, *14*, 709-718, doi:10.1111/j.0906-6705.2005.00349.x.
10. Kabza, M.; Karolak, J.A.; Rydzanicz, M.; Szcześniak, M.W.; Nowak, D.M.; Ginter-Matuszewska, B.; Polakowski, P.; Ploski, R.; Szaflik, J.P.; Gajecka, M. Collagen synthesis disruption and downregulation of core elements of TGF-β, Hippo, and Wnt pathways in keratoconus corneas. *Eur J Hum Genet* **2017**, *25*, 582-590, doi:10.1038/ejhg.2017.4.
11. Gozzelino, R.; Jeney, V.; Soares, M.P. Mechanisms of cell protection by heme oxygenase-1. *Annu Rev Pharmacol Toxicol* **2010**, *50*, 323-354, doi:10.1146/annurev.pharmtox.010909.105600.
12. Hua, X.; Deng, R.; Li, J.; Chi, W.; Su, Z.; Lin, J.; Pflugfelder, S.C.; Li, D.Q. Protective Effects of L-Carnitine Against Oxidative Injury by Hyperosmolarity in Human Corneal Epithelial Cells. *Invest Ophthalmol Vis Sci* **2015**, *56*, 5503-5511, doi:10.1167/iovs.14-16247.
13. Jalkanen, R.; Mäntyjärvi, M.; Tobias, R.; Isosomppi, J.; Sankila, E.M.; Alitalo, T.; Bech-Hansen, N.T. X linked cone-rod dystrophy, CORDX3, is caused by a mutation in the CACNA1F gene. *J Med Genet* **2006**, *43*, 699-704, doi:10.1136/jmg.2006.040741.
14. Cheng, H.; Liu, P.; Wang, Z.C.; Zou, L.; Santiago, S.; Garbitt, V.; Gjoerup, O.V.; Iglehart, J.D.; Miron, A.; Richardson, A.L.; et al. SIK1 couples LKB1 to p53-dependent anoikis and suppresses metastasis. *Sci Signal* **2009**, *2*, ra35, doi:10.1126/scisignal.2000369.
15. Consortium, T.A.o.G.R. Alliance of Genome Resources Portal: unified model organism research platform. *Nucleic Acids Research* **2019**, *48*, D650-D658, doi:10.1093/nar/gkz813.
16. Flower, D.R. The lipocalin protein family: structure and function. *Biochem J* **1996**, *318 (Pt 1)*, 1-14, doi:10.1042/bj3180001.
17. Hardcastle, A.J.; Liskova, P.; Bykhovskaya, Y.; McComish, B.J.; Davidson, A.E.; Inglehearn, C.F.; Li, X.; Choquet, H.; Habeeb, M.; Lucas, S.E.M.; et al. A multi-ethnic genome-wide association study implicates collagen matrix integrity and cell differentiation pathways in keratoconus. *Commun Biol* **2021**, *4*, 266.
18. Beaman, E.-M.; Carter, D.R.F.; Brooks, S.A. GALNTs: master regulators of metastasis-associated epithelial-mesenchymal transition (EMT)? *Glycobiology* **2022**, *32*, 556-579, doi:10.1093/glycob/cwac014.
19. Frausto, R.F.; Le, D.J.; Aldave, A.J. Transcriptomic Analysis of Cultured Corneal Endothelial Cells as a Validation for Their Use in Cell Replacement Therapy. *Cell Transplant* **2016**, *25*, 1159-1176, doi:10.3727/096368915x688948.
20. Fagerberg, L.; Hallström, B.M.; Oksvold, P.; Kampf, C.; Djureinovic, D.; Odeberg, J.; Habuka, M.; Tahmasebpoor, S.; Danielsson, A.; Edlund, K.; et al. Analysis of the human tissue-specific expression by genome-wide integration of transcriptomics and antibody-based proteomics. *Mol Cell Proteomics* **2014**, *13*, 397-406, doi:10.1074/mcp.M113.035600.
21. Meijers, R.; Smock, R.G.; Zhang, Y.; Wang, J.H. Netrin Synergizes Signaling and Adhesion through DCC. *Trends Biochem Sci* **2020**, *45*, 6-12, doi:10.1016/j.tibs.2019.10.005.
22. Vigouroux, R.J.; Cesar, Q.; Chédotal, A.; Nguyen-Ba-Charvet, K.T. Revisiting the role of Dcc in visual system development with a novel eye clearing method. *Elife* **2020**, *9*, doi:10.7554/eLife.51275.
23. Han, Y.; Shao, Y.; Lin, Z.; Qu, Y.-L.; Wang, H.; Zhou, Y.; Chen, W.; Chen, Y.; Chen, W.-L.; Hu, F.-R.; et al. Netrin-1 Simultaneously Suppresses Corneal Inflammation and

Neovascularization. *Investigative Ophthalmology & Visual Science* **2012**, *53*, 1285-1295, doi:10.1167/iovs.11-8722.
24. Andrysik, Z.; Bernstein, W.Z.; Deng, L.; Myer, D.L.; Li, Y.-Q.; Tischfield, J.A.; Stambrook, P.J.; Bahassi, E.M. The novel mouse Polo-like kinase 5 responds to DNA damage and localizes in the nucleolus. *Nucleic Acids Research* **2010**, *38*, 2931-2943, doi:10.1093/nar/gkq011.
25. Ito, Y.; Toriuchi, N.; Yoshitaka, T.; Ueno-Kudoh, H.; Sato, T.; Yokoyama, S.; Nishida, K.; Akimoto, T.; Takahashi, M.; Miyaki, S.; et al. The Mohawk homeobox gene is a critical regulator of tendon differentiation. *Proc Natl Acad Sci U S A* **2010**, *107*, 10538-10542, doi:10.1073/pnas.1000525107.
26. Fuest, M.; Yam, G.H.; Peh, G.S.; Mehta, J.S. Advances in corneal cell therapy. *Regen Med* **2016**, *11*, 601-615, doi:10.2217/rme-2016-0054.
27. Ito, Y.; Oike, Y.; Yasunaga, K.; Hamada, K.; Miyata, K.; Matsumoto, S.; Sugano, S.; Tanihara, H.; Masuho, Y.; Suda, T. Inhibition of angiogenesis and vascular leakiness by angiopoietin-related protein 4. *Cancer Res* **2003**, *63*, 6651-6657.
28. Chen, H.; Lui, Y.S.; Tan, Z.W.; Lee, J.Y.H.; Tan, N.S.; Tan, L.P. Migration and Phenotype Control of Human Dermal Fibroblasts by Electrospun Fibrous Substrates. *Adv Healthc Mater* **2019**, *8*, e1801378, doi:10.1002/adhm.201801378.
29. Chabannes, D.; Hill, M.; Merieau, E.; Rossignol, J.; Brion, R.; Soulillou, J.P.; Anegon, I.; Cuturi, M.C. A role for heme oxygenase-1 in the immunosuppressive effect of adult rat and human mesenchymal stem cells. *Blood* **2007**, *110*, 3691-3694, doi:10.1182/blood-2007-02-075481.
30. Jeon, K.I.; Kulkarni, A.; Woeller, C.F.; Phipps, R.P.; Sime, P.J.; Hindman, H.B.; Huxlin, K.R. Inhibitory effects of PPARγ ligands on TGF-β1-induced corneal myofibroblast transformation. *Am J Pathol* **2014**, *184*, 1429-1445, doi:10.1016/j.ajpath.2014.01.026.
31. Du, Y.; Sundarraj, N.; Funderburgh, M.L.; Harvey, S.A.; Birk, D.E.; Funderburgh, J.L. Secretion and organization of a cornea-like tissue in vitro by stem cells from human corneal stroma. *Invest Ophthalmol Vis Sci* **2007**, *48*, 5038-5045, doi:10.1167/iovs.07-0587.
32. Tanaka, Y.; Utsumi, J.; Matsui, M.; Sudo, T.; Nakamura, N.; Mutoh, M.; Kajita, A.; Sone, S.; Kigasawa, K.; Shibuya, M.; et al. Purification, molecular cloning, and expression of a novel growth-promoting factor for retinal pigment epithelial cells, REF-1/TFPI-2. *Invest Ophthalmol Vis Sci* **2004**, *45*, 245-252, doi:10.1167/iovs.03-0230.
33. Alles, V.V.; Bottazzi, B.; Peri, G.; Golay, J.; Introna, M.; Mantovani, A. Inducible expression of PTX3, a new member of the pentraxin family, in human mononuclear phagocytes. *Blood* **1994**, *84*, 3483-3493.
34. Jiang, Y.; Xing, X.; Niu, T.; Wang, H.; Wang, C.; Shi, X.; Liu, K.; Su, L. Protective effect of pentraxin 3 on pathological retinal angiogenesis in an in vitro model of diabetic retinopathy. *Arch Biochem Biophys* **2022**, *725*, 109283, doi:10.1016/j.abb.2022.109283.
35. Zhu, Y.T.; Li, F.; Zhang, Y.; Chen, S.Y.; Tighe, S.; Lin, S.Y.; Tseng, S.C.G. HC-HA/PTX3 Purified From Human Amniotic Membrane Reverts Human Corneal Fibroblasts and Myofibroblasts to Keratocytes by Activating BMP Signaling. *Invest Ophthalmol Vis Sci* **2020**, *61*, 62, doi:10.1167/iovs.61.5.62.
36. Doni, A.; Mantovani, A.; Bottazzi, B.; Russo, R.C. PTX3 Regulation of Inflammation, Hemostatic Response, Tissue Repair, and Resolution of Fibrosis Favors a Role in Limiting Idiopathic Pulmonary Fibrosis. *Front Immunol* **2021**, *12*, 676702, doi:10.3389/fimmu.2021.676702.

37. Peh, G.S.; Chng, Z.; Ang, H.P.; Cheng, T.Y.; Adnan, K.; Seah, X.Y.; George, B.L.; Toh, K.P.; Tan, D.T.; Yam, G.H.; et al. Propagation of human corneal endothelial cells: a novel dual media approach. *Cell Transplant* **2015**, *24*, 287-304, doi:10.3727/096368913x675719.
38. Alpha, K.M.; Xu, W.; Turner, C.E. Paxillin family of focal adhesion adaptor proteins and regulation of cancer cell invasion. *Int Rev Cell Mol Biol* **2020**, *355*, 1-52, doi:10.1016/bs.ircmb.2020.05.003.
39. Rush, J.S.; Boeving, M.A.; Berry, W.L.; Ceresa, B.P. Antagonizing c-Cbl Enhances EGFR-Dependent Corneal Epithelial Homeostasis. *Investigative Ophthalmology & Visual Science* **2014**, *55*, 4691-4699, doi:10.1167/iovs.14-14133.
40. Rosani, U.; Tarricone, E.; Venier, P.; Brun, P.; Deligianni, V.; Zuin, M.; Martines, E.; Leonardi, A.; Brun, P. Atmospheric-Pressure Cold Plasma Induces Transcriptional Changes in Ex Vivo Human Corneas. *PLoS One* **2015**, *10*, e0133173, doi:10.1371/journal.pone.0133173.
41. Sablina, A.A.; Hector, M.; Colpaert, N.; Hahn, W.C. Identification of PP2A complexes and pathways involved in cell transformation. *Cancer Res* **2010**, *70*, 10474-10484, doi:10.1158/0008-5472.Can-10-2855.
42. Clark, A.R.; Ohlmeyer, M. Protein phosphatase 2A as a therapeutic target in inflammation and neurodegeneration. *Pharmacol Ther* **2019**, *201*, 181-201, doi:10.1016/j.pharmthera.2019.05.016.
43. Liu, W.B.; Li, Y.; Zhang, L.; Chen, H.G.; Sun, S.; Liu, J.P.; Liu, Y.; Li, D.W. Differential expression of the catalytic subunits for PP-1 and PP-2A and the regulatory subunits for PP-2A in mouse eye. *Mol Vis* **2008**, *14*, 762-773.
44. Campbell, G.; Swamynathan, S.; Tiwari, A.; Swamynathan, S.K. The secreted Ly-6/uPAR related protein-1 (SLURP1) stabilizes epithelial cell junctions and suppresses TNF-α-induced cytokine production. *Biochem Biophys Res Commun* **2019**, *517*, 729-734, doi:10.1016/j.bbrc.2019.07.123.
45. Laux-Fenton, W.T.; Donaldson, P.J.; Kistler, J.; Green, C.R. Connexin expression patterns in the rat cornea: molecular evidence for communication compartments. *Cornea* **2003**, *22*, 457-464, doi:10.1097/00003226-200307000-00012.
46. Belinky, F.; Nativ, N.; Stelzer, G.; Zimmerman, S.; Iny Stein, T.; Safran, M.; Lancet, D. PathCards: multi-source consolidation of human biological pathways. *Database (Oxford)* **2015**, *2015*, doi:10.1093/database/bav006.
47. Collin, J.; Queen, R.; Zerti, D.; Bojic, S.; Dorgau, B.; Moyse, N.; Molina, M.M.; Yang, C.; Dey, S.; Reynolds, G.; et al. A single cell atlas of human cornea that defines its development, limbal progenitor cells and their interactions with the immune cells. *Ocul Surf* **2021**, *21*, 279-298, doi:10.1016/j.jtos.2021.03.010.
48. Ligocki, A.J.; Fury, W.; Gutierrez, C.; Adler, C.; Yang, T.; Ni, M.; Bai, Y.; Wei, Y.; Lehmann, G.L.; Romano, C. Molecular characteristics and spatial distribution of adult human corneal cell subtypes. *Scientific Reports* **2021**, *11*, 16323, doi:10.1038/s41598-021-94933-8.
49. VanderWall, K.B.; Lu, B.; Alfaro, J.S.; Allsop, A.R.; Carr, A.S.; Wang, S.; Meyer, J.S. Differential susceptibility of retinal ganglion cell subtypes in acute and chronic models of injury and disease. *Scientific Reports* **2020**, *10*, 17359, doi:10.1038/s41598-020-71460-6.
50. Yam, G.H.; Williams, G.P.; Setiawan, M.; Yusoff, N.Z.; Lee, X.W.; Htoon, H.M.; Zhou, L.; Fuest, M.; Mehta, J.S. Nerve regeneration by human corneal stromal keratocytes and stromal fibroblasts. *Sci Rep* **2017**, *7*, 45396, doi:10.1038/srep45396.

51. Yue, X.; Ai, J.; Xu, Y.; Chen, Y.; Huang, M.; Yang, X.; Hu, B.; Zhang, H.; He, C.; Yang, X.; et al. Polymeric immunoglobulin receptor promotes tumor growth in hepatocellular carcinoma. *Hepatology* **2017**, *65*, 1948-1962, doi:10.1002/hep.29036.
52. Zhang, Y.; Lu, W.; Chen, X.; Cao, Y.; Yang, Z. A Bioinformatic Analysis of Correlations between Polymeric Immunoglobulin Receptor (PIGR) and Liver Fibrosis Progression. *Biomed Res Int* **2021**, *2021*, 5541780, doi:10.1155/2021/5541780.
53. Morgan, R.O.; Fernandez, M.P. Expression profile and structural divergence of novel human annexin 31. *FEBS Lett* **1998**, *434*, 300-304, doi:10.1016/s0014-5793(98)00997-1.
54. Zhou, Y.; Qiu, C.; Wang, T.; Tao, L.; Zhang, Z.; Yao, J. High Expression of Annexin A9 Promotes Cell Proliferation and Migration in Gastric Cancer via the TGF-β Signaling Pathway. *J Environ Pathol Toxicol Oncol* **2021**, *40*, 87-94, doi:10.1615/JEnvironPatholToxicolOncol.2021038527.
55. Monastyrskaya, K.; Babiychuk, E.B.; Draeger, A. The annexins: spatial and temporal coordination of signaling events during cellular stress. *Cell Mol Life Sci* **2009**, *66*, 2623-2642, doi:10.1007/s00018-009-0027-1.
56. Wu, F.; Lee, S.; Schumacher, M.; Jun, A.; Chakravarti, S. Differential gene expression patterns of the developing and adult mouse cornea compared to the lens and tendon. *Exp Eye Res* **2008**, *87*, 214-225, doi:10.1016/j.exer.2008.06.001.
