# Supplementary figures and images for: Cytoprotective Effects of Human Platelet Lysate during the Xeno-Free Culture of Human Donor Corneas
Source: Int J Mol Sci. 2023 Feb 2;24(3):2882. doi: 10.3390/ijms24032882 (PMC9917909; doi:10.3390/ijms24032882)

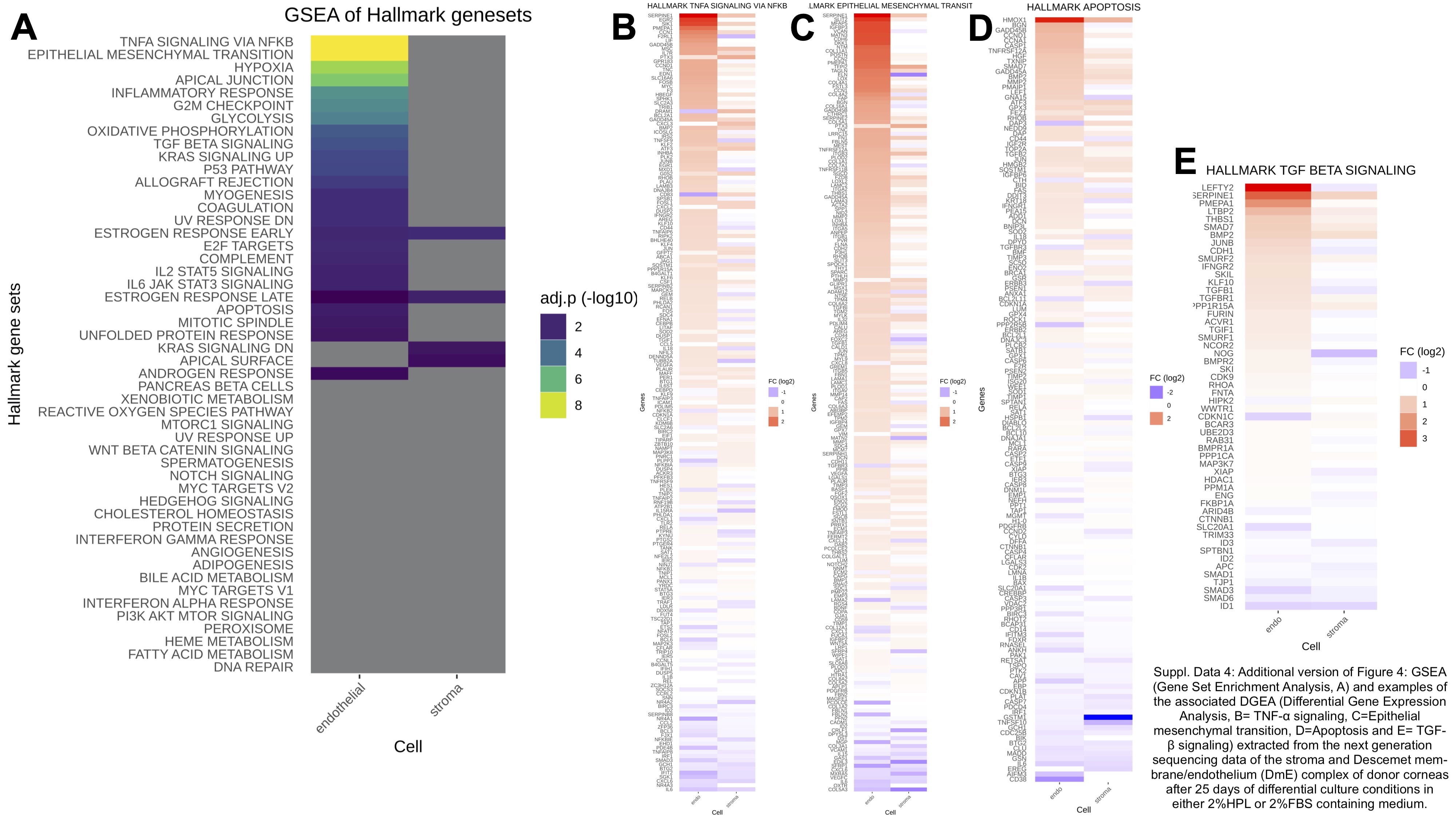

Supplement: Supplementary file 1 [file ijms-24-02882-s001.zip › supplementary Data S4 .jpg]
